# Screening and identification of a novel umami peptide with *in vitro* anti-inflammatory potential from sea cucumber protein: Integration of flavoromics, bioinformatic prediction, molecular simulation and experimental validation

**DOI:** 10.1016/j.fochx.2026.104580

**Published:** 2026-10-05

**Authors:** Zhongxing Chu, Can Long, Kangjie Xu, Xingjing Zhang, Dandan Qin, Zuomin Hu, Yaping Zhou, Feijun Luo, Qinlu Lin

**Affiliations:** a State Key Laboratory of Woody Oil Resources Utilization, School of Food Science and Engineering, Central South University of Forestry and Technology, Changsha 410004, Hunan, China; b DongTing Laboratory, Hunan Institute of Agricultural Product Processing and Quality Safety, Hunan Academy of Agricultural Sciences, Changsha, Hunan 410125, China

**Keywords:** Sea cucumber peptide, Umami peptide, Anti-inflammatory potential, Flavoromics, Molecular simulation

## Abstract

Sea cucumber protein was sequentially hydrolyzed with pepsin, trypsin, and enterokinase to obtain peptides with desirable taste characteristics and immunomodulatory potential. Multi-scale characterization, flavoromics, peptidomics, bioinformatic screening, molecular simulations, and cellular assays were employed to investigate the structural changes, flavor remodeling, and bioactivity of the resulting hydrolysates. Results showed that hydrolysis disrupted the compact protein structure, induced conformational unfolding, and yielded short peptides (0.5–1.5 kDa) with enhanced hydrophilicity and processing adaptability. The volatile profile was significantly reshaped, with marked enrichment of aldehydes and heterocyclic compounds. Through multi-dimensional screening, the pentapeptide FDRGF (TH) was identified with outstanding umami taste and low bitterness. Molecular docking and dynamics simulations confirmed stable binding of TH to immunomodulatory targets, and cellular assays verified that TH improved macrophage metabolic status and regulated the expression of inflammatory mediators under LPS-induced inflammatory conditions. This study elucidates the structure-flavor-activity relationships of sea cucumber protein hydrolysates and identifies a dual-function umami peptide with immunomodulatory activity, providing theoretical support for the value-added utilization of sea cucumber resources.

## Highlights

• FDRGF exhibits favorable umami taste and *in vitro* anti-inflammatory potential.
• Multi-enzyme hydrolysis remodels structure and volatile flavor of sea cucumber protein.
• FDRGF mitigates inflammation *via* suppressing ROS-NF-κB signaling in macrophages.

## 1 Introduction

Sea cucumber is a traditional high-value seafood product in China and an important marine biological resource globally, with China's sea cucumber aquaculture and processing output consistently ranking among the highest in the world. The annual aquaculture yield of fresh sea cucumber in China has exceeded 220,000 tons according to *China Fishery Statistical Yearbook*, generating abundant raw-protein resources for deep-processing exploitation (Dou et al., 2026). Sea cucumber is rich in high-quality collagen, polysaccharides, and various bioactive components, and has long been recognized as a nutritional food with both medicinal and edible values (Harini et al., 2024; Hidayah et al., 2026). As the marine food industry transitions toward deep processing, conventional primary processing methods such as rehydration and drying can no longer meet the demands for high-value utilization. Therefore, how to fully unleash the nutritional and functional values of sea cucumber protein through modern biotechnology and develop high value-added food ingredients has become a research hotspot in the field of sea cucumber processing.

Bioactive peptides are small-molecule peptides derived from proteins through enzymatic hydrolysis or separation and purification. Owing to the low molecular weight, easy digestibility and absorption, mild physiological activities, and high safety, they exhibit broad application prospects in functional foods, nutritional supplements, and special dietary products (Lu et al., 2022; Yao et al., 2026). Among the various types of bioactivities, immunomodulatory activity is one of the most concerned core functions of food-derived peptides. Unlike pharmaceutical interventions, food-derived immunomodulatory peptides primarily exert nutritional regulatory effects by modulating the physiological status of innate immune cells, maintaining immune homeostasis, and enhancing the body's defense capacity against external stimuli, which aligns with the functional positioning of food bioactive ingredients (Nikoo et al., 2026; Zhang et al., 2025). Previous studies have confirmed that various marine-derived protein peptides possess immunomodulatory potential. However, research on sea cucumber-derived peptides has mostly focused on the activity validation of crude extracts, while systematic analysis of the precise identification, structure-activity relationships, and mechanisms of action of characteristic peptides remains relatively insufficient.

Flavor quality is a core sensory attribute that determines the market acceptance and application scenarios of food ingredients, and is also a critical factor that cannot be overlooked in peptide product development (Zhang et al., 2024). Enzymatic proteolysis is the core technology for preparing active peptides. During enzymatic hydrolysis, while cleaving peptide bonds and releasing active peptide fragments, the process also disrupts the original matrix structure of the raw material, promoting a series of chemical reactions such as lipid oxidation and Maillard reaction, thereby reshaping the flavor profile of the product from both volatile aroma and taste dimensions (Ashaolu et al., 2024; Sun & Udenigwe, 2020). On the one hand, moderate enzymatic hydrolysis can release taste-active amino acids and umami peptides, improving the flavor complexity of the product. On the other hand, insufficient or excessive hydrolysis may introduce sensory defects such as bitterness and fishy odor (Chen et al., 2026). In recent years, the rapid development of high-resolution flavoromics technology based on GC-Orbitrap/MS and electronic tongue taste analysis has provided powerful tools for systematically identifying flavor defects and beneficial taste compounds during enzymatic hydrolysis, as well as for precisely targeting characteristic taste peptides (Sospeter & Ding, 2026). However, current studies on sea cucumber peptides have mostly treated flavor as a subsidiary indicator with only brief descriptions, and few have elucidated the regulatory mechanisms of enzymatic hydrolysis on flavor formation from the perspective of protein structural evolution. Moreover, targeted peptide screening that simultaneously considers both taste characteristics and immunomodulatory activity is even rarer, which has constrained the industrial application of sea cucumber peptides as high-quality flavor-functional ingredients.

In this context, the present study utilized sea cucumber protein as raw material and employed a sequential enzymatic hydrolysis process with pepsin, trypsin, and enterokinase to prepare sea cucumber peptides. Scanning electron microscopy (SEM), Fourier-transform infrared spectroscopy (FTIR), ultraviolet-visible spectroscopy (UV–Vis), intrinsic fluorescence spectroscopy, X-ray diffraction (XRD), and differential scanning calorimetry (DSC) were combined to characterize the differences of microscopic morphology, molecular conformation, aggregation state and thermal stability between native sea-cucumber protein and hydrolysate obtained under this fixed multi-enzyme hydrolysis condition. HS-SPME-GC-Orbitrap/MS was employed for comprehensive flavoromics analysis, coupled with multivariate statistical methods to identify the differences and evolution patterns of volatile flavor compounds before and after hydrolysis. Peptide sequences were precisely identified by LC-MS/MS, and bioinformatics tools were applied for multi-dimensional screening of candidate peptides based on taste characteristics, immunomodulatory activity, and safety properties, with taste characteristics further validated by electronic tongue analysis. Molecular docking and molecular dynamics simulations were conducted to preliminarily explore the interaction mechanisms between the characteristic peptide and immune targets, and the immunomodulatory activity was verified using a RAW 264.7 macrophage model. This study aimed to unravel the inter-connections among protein structural alteration, volatile-flavor remodeling and peptide-derived bioactivity under this specific multi-enzyme hydrolysis condition, and to identify sea cucumber-derived characteristic peptides with both excellent umami taste and immunomodulatory activity, thereby providing theoretical basis for the high-value deep processing of sea cucumber resources and the development of flavor-functional peptides.

## 2 Materials and methods

### 2.1 Experimental reagents

Sea cucumber protein (protein purity >90%; moisture: 6.2 ± 0.3%; collagen content >82%) was provided by Baidefu Biotechnology Co., Ltd. (Tangshan, Hebei, China). Trypsin (Cat. P00761, ≥250 USP U/mg), pepsin (Cat. P00791, ≥10,000 U/g), and enterokinase (Cat. R141088, ≥5 U/μl) were purchased from Aladdin Biochemical Technology Co., Ltd. (Shanghai, China). Synthetic pentapeptide TH (FDRGF, purity >98%) was prepared *via* solid-phase peptide synthesis by Gill Biochemical Co., Ltd. (Shanghai, China). All cell-related assay kits were obtained from Beyotime Biotechnology (Shanghai, China). Other chemical reagents used were of analytical grade.

### 2.2 Preparation of sea cucumber peptides

In the present work, the sequential multi-enzyme hydrolysis process was designed for targeted preparation of umami and immunomodulatory peptides from sea cucumber protein, which differs from conventional *in vitro* gastrointestinal simulated digestion. Pepsin, active trypsin, and enterokinase were applied sequentially for the hydrolysis of sea cucumber protein. Although enterokinase is well-known for its physiological role in activating trypsinogen, previous reports have indicated that enterokinase can also exert limited endoproteolytic activity toward specific basic residue-rich sites in collagen-rich protein substrates. The substrate was mixed with water at a ratio of 1:5 (w/v), and the three proteases (pepsin, trypsin, enterokinase) were introduced successively with an enzyme-to-substrate mass ratio of 1% (w/w). Pepsin-catalyzed hydrolysis was performed at pH 2.0, while trypsin and enterokinase digestions were carried out at pH 8.0; all enzymatic reactions were conducted at 37 °C for 4 h. After each hydrolysis stage, enzymes were heat-inactivated to terminate proteolysis. A blank control without exogenous protease supplementation was processed in parallel under identical temperature, pH and incubation duration to eliminate interference originating from endogenous proteases in raw materials. The reaction mixtures were centrifuged at 13000 ×*g* for 20–25 min, supernatants were collected, lyophilized and stored at −20 °C for subsequent analysis.

The degree of hydrolysis (DH) of the final hydrolysate was determined by the o-phthalaldehyde (OPA) colorimetric method with slight modifications (Chu et al., 2025). Briefly, an aliquot of hydrolysate was mixed with an equal volume of 20% (w/v) trichloroacetic acid (TCA) and stood at room temperature for 30 min to precipitate undigested protein. After centrifugation (10,000 ×*g*, 15 min), 200 μL of the supernatant was mixed with 4 mL of freshly prepared OPA reagent and incubated in the dark at room temperature for 2 min. Absorbance was measured at 340 nm, and L-leucine was used as the standard for quantifying free α-amino groups. The total α-amino group content of the native sea cucumber protein was determined after complete acid hydrolysis (6 mol/L HCl, 110 °C, 24 h). DH was calculated as follows:

DH (%) = (α-amino groups in TCA supernatant / total α-amino groups of native protein) × 100.

### 2.3 Scanning electron microscopy (SEM)

Lyophilized sea cucumber protein and peptide powder samples were uniformly adhered to the surface of scanning electron microscope specimen holders using conductive adhesive tape. Loose powder that was not firmly attached was gently blown off with a rubber ear bulb to avoid surface stacking. To improve electrical conductivity and reduce charge-induced image drift and artifacts, the specimens were coated with a gold layer using an ion sputtering device prior to observation. SEM observation was then performed at a suitable accelerating voltage to examine the surface morphology of the samples. Micrographs at different magnifications were obtained to compare the differences in particle morphology, surface roughness, pore structure, and particle aggregation states between sea cucumber protein and sea cucumber peptide samples.

### 2.4 Fourier-transform infrared (FTIR) spectroscopy

For FTIR analysis, lyophilized protein and peptide samples were individually blended with spectroscopic-grade KBr at a ratio of 1:100 (w/w). The mixtures were finely ground in an agate mortar under infrared irradiation to reduce moisture interference. After thorough homogenization, the ground mixtures were pressed into transparent disks. Spectral data were acquired on a Fourier-transform infrared spectrometer across the range of 4000–500 cm^−1^, with a resolution of 4 cm^−1^ and 32 co-added scans. A blank KBr disk served as the background, and its spectrum was subtracted from each sample spectrum. The resulting spectra were then used to assess the secondary structural changes induced by enzymatic hydrolysis of sea cucumber protein.

### 2.5 UV–vis spectroscopy and intrinsic fluorescence spectroscopy

Lyophilized sea-cucumber peptide powder was fully dissolved in ultrapure water to prepare 1 mg−/mL solution. For native sea-cucumber protein (poor water-solubility), 1 mg/mL suspension was prepared by dispersing lyophilized protein powder in ultrapure water under continuous magnetic stirring. Absorption spectra were then recorded on a UV–Vis spectrophotometer over 200–400 nm, with ultrapure water serving as the blank. For intrinsic fluorescence measurement, the same 1 mg/mL solutions were used. Emission spectra were collected from 300 to 450 nm with an excitation wavelength of 280 nm and a slit width of 5 nm. The maximum emission wavelength and corresponding fluorescence intensity were recorded for each sample to evaluate conformational differences between the protein and peptide groups.

### 2.6 X-ray diffraction (XRD) measurement

Lyophilized sea cucumber protein and peptide powders were evenly spread onto the sample holder of an X-ray diffractometer and pressed flat to ensure the sample surface was flush with the reference plane of the holder. XRD analysis was performed under the following conditions: Cu Kα radiation (λ = 1.5406 Å), 2θ scanning range of 5°-90°, and scanning rate of 4°/min. The XRD diffraction patterns of the two groups of samples were collected, and the positions, shapes, and intensities of diffraction peaks were compared to analyze the changes in crystallinity and amorphous structure of sea cucumber protein before and after enzymatic hydrolysis.

### 2.7 Differential scanning calorimetry (DSC) thermodynamic measurement

Accurately weighed portions (20 mg) of lyophilized sea cucumber protein and peptide powders were placed into aluminum crucibles and hermetically sealed by pressing. An empty crucible was used as the reference. Thermal measurements were conducted under a nitrogen flow of 50 mL/min, with the temperature increased from 25 to 200 °C at 10 °C/min. The resulting heat flow–temperature curves were recorded, from which thermodynamic parameters including denaturation temperature (Tm) and enthalpy change (ΔH) were derived to assess the impact of enzymatic hydrolysis on the thermal behavior of sea cucumber protein and its derived peptides.

### 2.8 Flavoromics analysis of sea cucumber peptides

Volatile compounds were extracted and analyzed by HS-SPME coupled with GC-Orbitrap/MS, following a previously reported procedure with slight adjustments. Briefly, 1.0 g of each lyophilized sample (protein or peptide) was combined with 5 mL of saturated NaCl and 20 μL of internal standard (10 μg/mL). A 120 μm DVB/CARBON/PDMS fiber was used for extraction at 60 °C, and the adsorbed volatiles were desorbed at 260 °C. Chromatographic separation was achieved on a TG-WAXMS capillary column, and mass detection was performed in EI full-scan mode (*m*/*z* 35–450). Compound identification was carried out by baseline correction, spectral deconvolution, and NIST library searching. Relative contents were determined *via* the internal standard method, and relative odor activity values (rOAV) were computed to assess aroma contributions. Only compounds with RSD < 0.5 in QC samples were kept to ensure data reliability. Multivariate analyses, including PCA and OPLS-DA, were conducted with 200 permutation tests to avoid overfitting. Differential volatiles were filtered using VIP ≥ 1, *P* < 0.05, and FC > 1.5 or < 0.67, while non-contributing compounds were removed based on a self-compiled list of odor-active substances. Final data visualization was accomplished using R and Python-Plotly.

### 2.9 Identification of sea cucumber peptides

Prior to LC-MS/MS analysis, sea cucumber peptide samples were subjected to desalting using C18 cartridges. The cartridges were successively rinsed with 100% acetonitrile, equilibrated with 0.1% formic acid, washed with the same acid solution to remove contaminants, and finally eluted with 70% acetonitrile to recover the peptide fractions. The eluates were then lyophilized, and the dried residues were re-dissolved in 20.0 μL of 0.1% formic acid, from which 3.0 μL aliquots were injected *via* the autosampler. Peptide separation and identification were carried out on an LC-MS/MS system equipped with an analytical column at a flow rate of 500 nL/min. The acquired raw MS data were processed using MaxQuant (v1.6.10.43) and FragPipe. Peptide-spectrum matches were searched against the Holothuroidea (sea cucumber) protein database downloaded from NCBI RefSeq database. Following database searching, peptide quantification was accomplished using the iBAQ algorithm implemented in MaxQuant.

### 2.10 Functional prediction of sea cucumber peptides

It is well established that bioactive peptides often share functional attributes with their parent proteins. Accordingly, to gain insight into the potential activities of the identified peptides, GO and KEGG pathway enrichment analyses were carried out on the corresponding precursor proteins. Based on the enrichment outcomes, several public databases-including Targetnet (http://targetnet.scbdd.com/calcnet/index/), SEA (http://sea.bkslab.org/), OMIM (https://omim.org/), and DigSee (http://210.107.182.61/geneSearch/)-were employed for target screening, and the resulting data were visualized using R software.

### 2.11 Screening of sea cucumber peptides

Following peptide identification by LC-MS/MS, the resulting sequence data were standardized in format. Umami and bitterness potentials were predicted using the online tools available at https://pmlabstack.pythonanywhere.com/predict_umami and https://pmlab.pythonanywhere.com/bitterbertpredict, respectively. The PeptideRanker server (http://distilldeep.ucd.ie/PeptideRanker/) was employed to evaluate the overall bioactivity likelihood of each peptide based on its ranking score. Immunomodulatory potential was assessed *via* the CSM-peptides platform (https://biosig.lab.uq.edu.au/csm_peptides/), while water solubility was estimated using the Innovagen tool (http://www.innovagen.com/proteomics-tools). In addition, toxicity was evaluated with ToxinPred (https://webs.iiitd.edu.in/raghava/toxinpred/), and allergenic risk was examined using the AllerTOP v2.1 server (https://mybiosoftware.com).

### 2.12 Molecular docking and molecular dynamics simulation

To evaluate the interaction between peptide TH and its potential targets, molecular docking was carried out with the selected target proteins as receptors and TH as the ligand. Binding free energies were calculated to assess the affinity of TH for each target. To further verify the conformational stability of the docked complexes and to characterize the dynamic binding behavior, molecular dynamics (MD) simulations were subsequently performed using GROMACS 2020.6 over a total simulation period of 100 ns. The receptor proteins were modeled with the AMBER99SB-ILDN force field, while topology parameters for TH were generated *via* Amber20 in conjunction with the GAFF force field. Each complex was solvated in a cubic TIP3P water box with a 10 nm edge length, and Na^+^/Cl^−^ ions were added as needed to achieve charge neutrality. The simulation protocol comprised energy minimization, 100 ps NVT equilibration at 300 K, and 100 ps NPT equilibration at 300 K and 101.325 kPa. The final production runs were conducted under the NPT ensemble, with temperature regulated by the V-rescale thermostat and pressure controlled by the Parrinello-Rahman barostat.

### 2.13 Electronic tongue analysis

Sea cucumber peptide samples were redissolved in ultrapure water at the same solid concentration. After complete dissolution and equilibration at room temperature, the supernatant was collected for analysis. Taste attributes including bitterness, umami, saltiness, sweetness, and astringency were measured using an electronic tongue system (ASTREE II, Alpha MOS, France). Each sample was measured six times in parallel, with the first two measurements discarded to eliminate system equilibration effects, and the remaining four sets of data were used for statistical analysis. The response signals of each sensor were collected, and taste response values were output. Multivariate statistical analysis was performed using SIMCA software (version 14.1, Umetrics, Sweden). Principal component analysis (PCA) was conducted to examine the clustering and discrimination of different sea cucumber peptide samples and reference compounds (MSG, GSH), and score plots with 95% confidence intervals (*n* = 4 per group) were generated.

### 2.14 Immunomodulatory activity validation of sea cucumber peptide TH

RAW 264.7 macrophages at logarithmic growth phase were harvested by trypsinization, resuspended, and adjusted to 5 × 10^5^ cells/mL. For the MTS assay, cells were dispensed into 96-well plates (100 μL/well) and incubated at 37 °C with 5% CO₂ until attachment. The culture medium was then replaced with fresh medium supplemented with TH at varying concentrations, with triplicate wells for each condition. Following 24 h of exposure, 10 μL of MTS solution was added to each well. After a 30-min dark incubation and 10-min gentle shaking on an orbital shaker, absorbance was read at 490 nm using a microplate reader, and cell viability was determined following the manufacturer's protocol. For morphological and nuclear staining as well as ROS measurement, cells were seeded into 6-well plates (2 mL/well, 5 × 10^5^ cells/mL). After adhesion, the cells were pre-incubated with different concentrations of TH for 2 h and subsequently stimulated with 2 ‰ (*v*/v) lipopolysaccharide (LPS, stock solution: 1 mg/mL in sterile phosphate-buffered saline) to induce an inflammatory response. For H&E staining, the cells were rinsed with PBS, fixed in 4% paraformaldehyde for 10–15 min at ambient temperature, stained with hematoxylin for 5–10 min, differentiated, and counterstained with eosin for 30 s to 2 min. Morphological changes were documented under an inverted fluorescence microscope. For Hoechst 33342 staining, the fixed cells were permeabilized with 0.1% Triton X-100 and incubated with Hoechst 33342 solution in the dark for 30 min at room temperature. Hoechst 33342 (Cat: IYT3223) was provided by Beijing Solarbio Science & Technology Co., Ltd. After PBS washing, nuclear morphology was visualized and imaged. Intracellular ROS levels were evaluated using the DCFH-DA probe: cells were incubated with the probe working solution at 37 °C in the dark for 20 min, washed thoroughly to eliminate excess probe, and fluorescence intensity was recorded on a microplate reader.

### 2.15 Data processing and statistical analysis

All graphical representations were created with GraphPad Prism. Statistical evaluations were carried out using SPSS 22.0. Inter-group comparisons were assessed by one-way analysis of variance (ANOVA), followed by suitable post-hoc tests, and statistical significance was set at *P* < 0.05. All experimental data were expressed as mean ± standard deviation (SD) based on a minimum of three independent replicates.

## 3 Results and discussion

### 3.1 Basic hydrolysis degree of sea cucumber protein hydrolysates

The DH of sea-cucumber protein after sequential pepsin-trypsin-enterokinase hydrolysis was quantified using the OPA colorimetric method. The DH value of final hydrolysate reached 40.72 ± 1.19%. This value demonstrated that the sequential multi-enzyme treatment effectively cleaved peptide bonds of macromolecular sea-cucumber collagen protein, generating large amounts of peptide fragments. This observation provides the foundational evidence to support the subsequent structural change, flavor remodeling and bio-peptide identification results described below. Nevertheless, only the hydrolysis degree was measured in this study to reflect peptide-bond breaking efficiency, while peptide yield, soluble protein and soluble peptide recovery were not determined. Hence, quantitative evaluation on substrate-to-target-peptide conversion efficiency cannot be achieved at the current stage. Further research will supplement these indicators and explore their correlations with flavor characteristics and immunomodulatory activity of hydrolysates.

### 3.2 Microscopic morphology observation of sea cucumber peptides

The microstructures of sea cucumber protein and sea cucumber peptides were observed by scanning electron microscopy (SEM) (Fig. 1). At 100× magnification, sea cucumber protein appeared as irregular sheet-like and block-like aggregates with sharp edges, a dense structure, and relatively smooth surfaces, displaying typical characteristics of unhydrolyzed macromolecular polymers. At 1000× magnification, the surface of sea cucumber protein exhibited layered or torn textures, with no obvious pore structures, indicating strong intermolecular forces that formed a continuous solid matrix. In contrast, the microscopic morphology of sea cucumber peptides changed markedly. At 100× magnification, the sample surface exhibited obvious porous structures with numerous spherical or irregular pores of various sizes. At 1000× magnification, sea cucumber peptides formed a network-like or sponge-like structure with abundant cavities, thick pore walls, and interconnected pores. This porous and rough surface morphology is primarily attributed to the cleavage of macromolecular peptide bonds by proteases, which disrupted the original compact spatial structure, while the release of small molecules and gas evolution during hydrolysis promoted the formation of new channels.

**Fig. 1:**
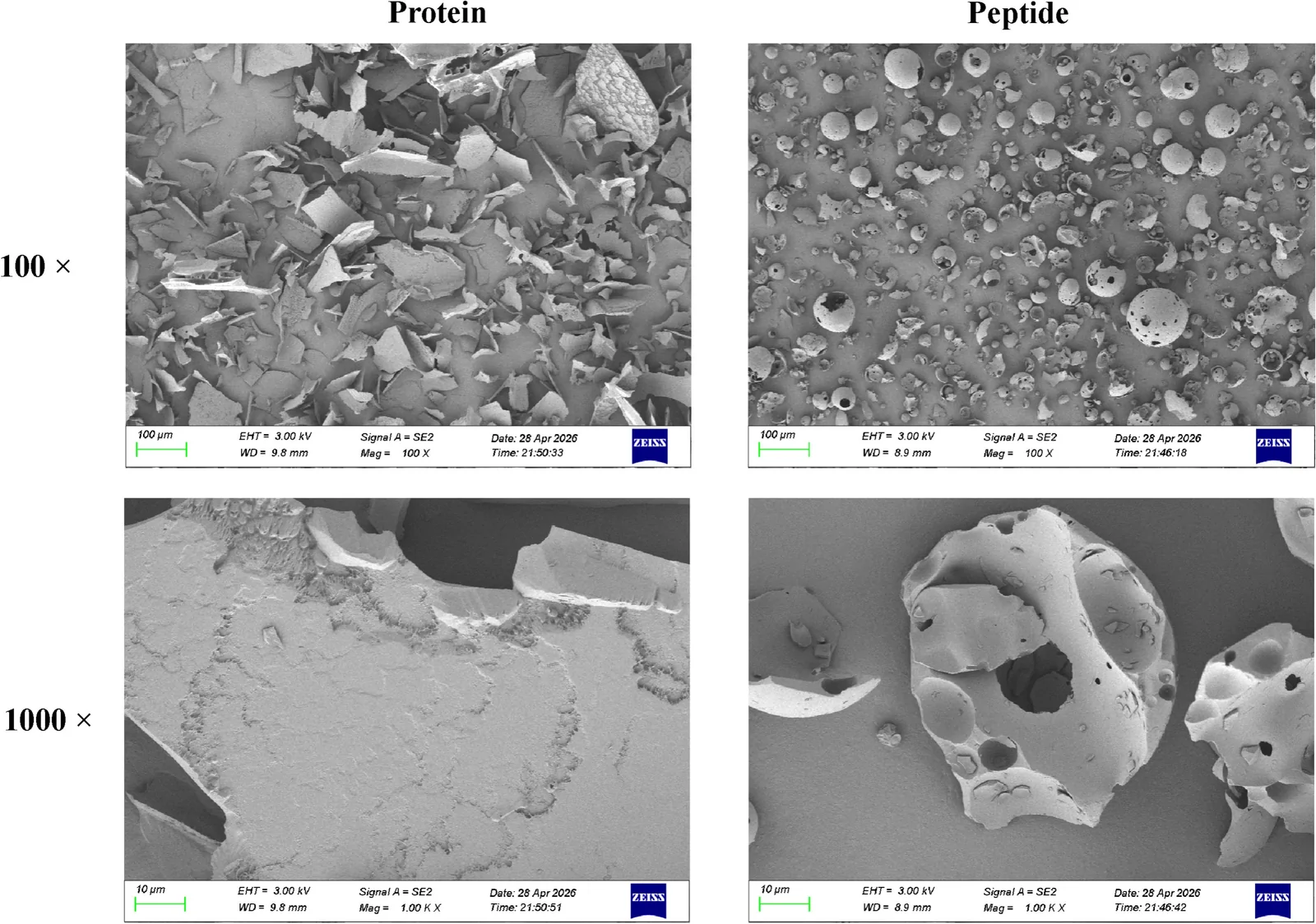
Scanning electron microscopy (SEM) images of sea cucumber protein (Protein) and sea cucumber peptide hydrolysates (Peptide) at different magnifications (100× and 1000×).

Enzymatic hydrolysis is the primary cause of the dramatic changes in the microscopic morphology of sea cucumber protein. Proteases specifically cleave peptide bonds, disrupting non-covalent forces such as hydrogen bonds and hydrophobic interactions that maintain the higher-order structure of proteins, leading to the disintegration of the compact three-dimensional network and the breakdown of macromolecular proteins into small peptide fragments (Liu et al., 2025). This process is accompanied by a decrease in mass-to-volume ratio and an increase in specific surface area, resulting in the formation of the loose porous structure observed in the micrographs.

### 3.3 FTIR analysis

As shown in Fig. 2A, FTIR spectroscopy was used to analyze the functional groups and secondary structure changes of the samples. Both the protein and peptide groups exhibited broad absorption peaks near 3400 cm^−1^, attributed to O—H and N—H stretching vibrations, with the peptide group showing broader peak shapes, suggesting changes in the hydrogen bonding network. In the 1600–1700 cm^−1^ region (amide I band, highlighted in yellow), the protein group displayed strong absorption near 1650 cm^−1^, corresponding to α-helix and random coil structures. In contrast, the peptide group showed obvious peak splitting and shifting in this region, with significantly enhanced absorption intensity near 1540 cm^−1^ (amide II band).

**Fig. 2:**
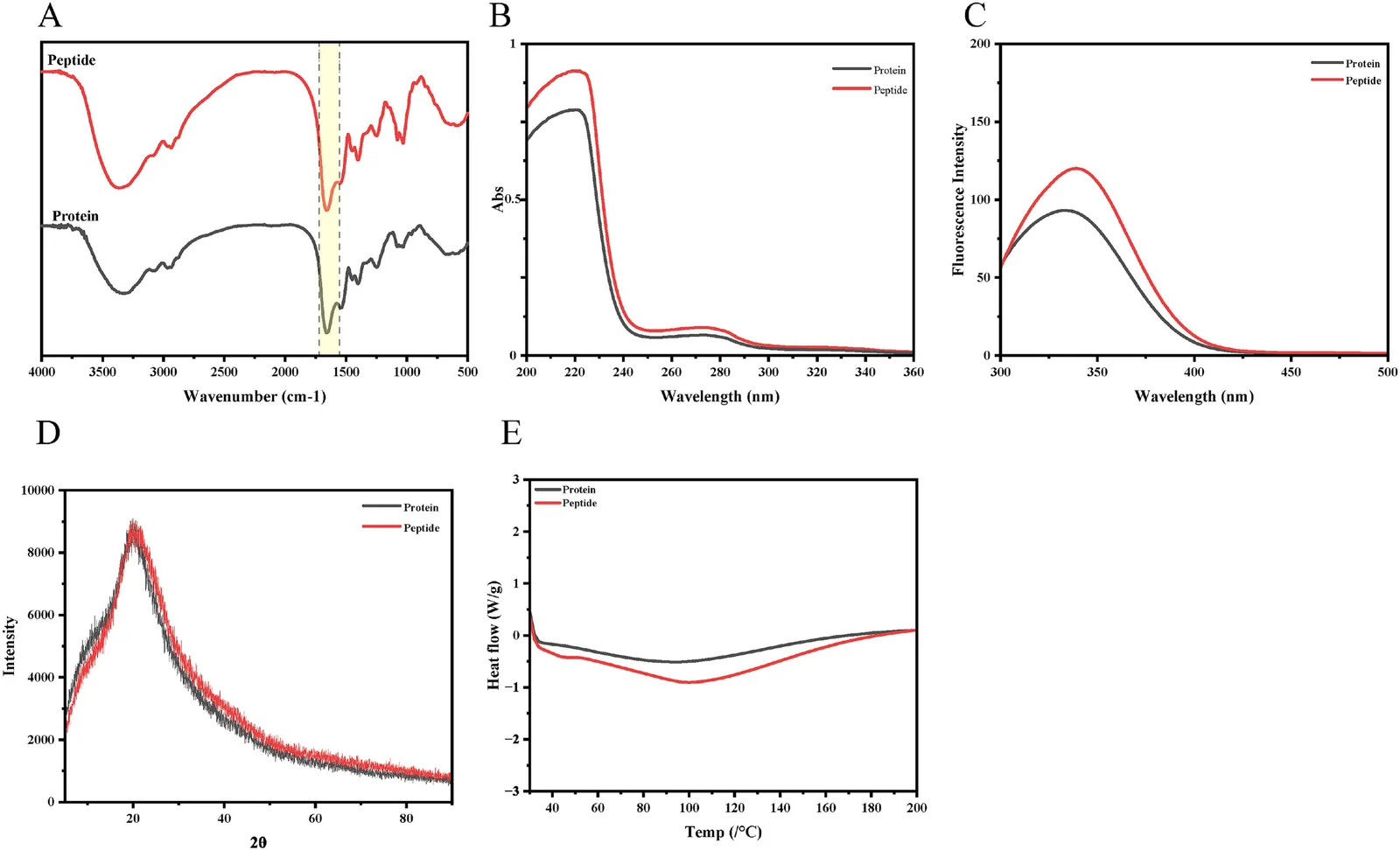
Structural characterization of sea cucumber protein and its hydrolysates. (A) FTIR spectra; (B) UV–Vis absorption spectra; (C) Intrinsic fluorescence spectra; (D) XRD patterns; (E) DSC thermograms.

The amide I band is the most sensitive region for probing protein secondary structure, and its peak shifts and splitting directly reflect changes in secondary structure composition: after enzymatic hydrolysis, the proportion of ordered structures such as α-helix decreased, while the proportion of disordered structures such as random coil increased (Maurya et al., 2026). The underlying mechanism is that the α-helical conformation of proteins is highly dependent on intra-chain and inter-chain hydrogen bonding networks. After peptide bond cleavage, the original hydrogen bond equilibrium is disrupted, causing the ordered helices to lose their support and undergo depolymerization, ultimately converting to random coil conformations (Xiang et al., 2025). The absorption at 1540 cm^−1^ corresponds to the amide II band characteristic vibration of peptide bonds. The enhanced absorption in this region results from the increased total number of peptide bonds per unit mass after hydrolysis-after macromolecules are cleaved into numerous short peptides, the peptide bond density per unit mass increases significantly (Liang et al., 2026).

### 3.4 UV–vis absorption spectroscopy analysis

As shown in Fig. 2B, UV–Vis absorption spectroscopy was used to detect the presence and microenvironmental changes of aromatic amino acids (tryptophan, tyrosine, and phenylalanine) in proteins/peptides. The protein group exhibited a distinct peptide bond absorption peak at 220 nm and a weak absorption at 280 nm due to the presence of aromatic amino acid residues. In contrast, the peptide group showed significantly increased absorbance at 220 nm, and the absorption peak at 280 nm also exhibited a hyperchromic effect.

The significant increase in absorbance at 220 nm arises from the higher total number of peptide bonds per unit mass after hydrolysis, along with changes in molecular concentration or chain rigidity. The enhanced absorbance at 280 nm suggests that enzymatic hydrolysis induced polypeptide chain unfolding, exposing aromatic amino acid residues that were originally buried in the hydrophobic core to the polar environment. This change in the microenvironment increased the hydrophilicity of the polypeptide chains, providing a chemical basis for subsequent improvements in solubility (Nain et al., 2026).

### 3.5 Intrinsic fluorescence spectroscopy analysis

As shown in Fig. 2C, intrinsic fluorescence spectroscopy further reflects the degree of tertiary structure unfolding by monitoring the fluorescence responses of tryptophan (Trp) and tyrosine (Tyr) residues. The protein group exhibited a fluorescence emission peak at approximately 330 nm, which is characteristic of Trp residues in a hydrophobic environment. After enzymatic hydrolysis, the fluorescence intensity of the peptide group significantly increased (hyperchromic effect), and the emission peak underwent a red shift, with the maximum shifting to approximately 345 nm.

The red shift of the emission peak is a reliable indicator of increased environmental polarity around Trp residues, confirming that enzymatic hydrolysis disrupted the tertiary structure and induced polypeptide unfolding, exposing Trp residues from the hydrophobic core to the aqueous environment. The increase in fluorescence intensity may be attributed to reduced intermolecular quenching after hydrolysis, and is regarded as an auxiliary indicator rather than direct evidence of unfolding. This unfolding process not only improved the solubility of the peptides but also increased molecular flexibility and exposed active sites, facilitating their absorption and utilization in biological systems (Wang et al., 2025).

### 3.6 XRD analysis

As shown in Fig. 2D, XRD patterns were used to analyze the crystallinity and aggregation state of the samples. Both the protein and peptide groups exhibited a broad diffuse peak at 2θ ≈ 20°, indicating that both belong to amorphous structures. However, the peptide group displayed significantly higher diffraction peak intensity at this position with a sharper peak shape. This suggests that after enzymatic hydrolysis, the short peptide molecules may undergo intermolecular hydrogen bond rearrangement during drying, forming a higher degree of short-range ordered arrangement, although contributions from sample particles and preparation factors to the diffraction signal cannot be excluded (Sun et al., 2026). Considering the characteristics of small-molecule peptides, this microstructural ordering may result from the tendency of short-chain peptides to form dense aggregates through hydrogen bonding during drying, which also explains their favorable powder forming properties.

### 3.7 DSC analysis

As shown in Fig. 2E, DSC curves were used to evaluate the thermal stability of the samples. The protein group exhibited an obvious endothermic peak near 100 °C, corresponding to the denaturation temperature (Tm) of the protein. In contrast, the peptide group showed not only significantly reduced endothermic peak intensity but also broader peak shapes, with the Tm value slightly decreased or even disappearing.

The disappearance or weakening of the protein denaturation endothermic peak indicates that enzymatic hydrolysis degraded macromolecular proteins into small-molecule peptides, destroying the intact tertiary structure that maintains thermal stability and resulting in a significant decrease in enthalpy change (ΔH). Small-molecule peptide segments have higher thermal motion freedom and no longer exhibit the cooperative thermal denaturation behavior characteristic of intact proteins (Pang et al., 2025). This reduced thermal stability suggests that sea cucumber peptides are less prone to coagulation or precipitation during processing, demonstrating good processing adaptability.

### 3.8 Comprehensive flavoromics analysis of sea cucumber peptides

Comprehensive flavoromics analysis revealed a significant reshaping of the volatile compound profile of sea cucumber following enzymatic hydrolysis. The PCA score plot (Fig. 3A) showed complete separation between the sea cucumber protein and peptide groups in PC space (with significant cumulative variance contribution from PC1 and PC2), indicating that enzymatic hydrolysis induced a fundamental transformation of the flavor profile. Compound classification statistics (Fig. 3B) showed that the relative abundances or numbers of aldehydes, alcohols, and heterocyclic compounds in the peptide group significantly increased compared to the protein group, with aldehydes becoming the major flavor contributors in the peptide group. Radar plots (Fig. 3C) further visualized the differences in sensory attributes: compared with the protein group, sea cucumber peptides showed significantly enhanced response intensities in dimensions such as “fatty,” “green/grassy,” “fried,” and “waxy,” while the protein group was dominated by “earthy” and “fresh” notes.

**Fig. 3:**
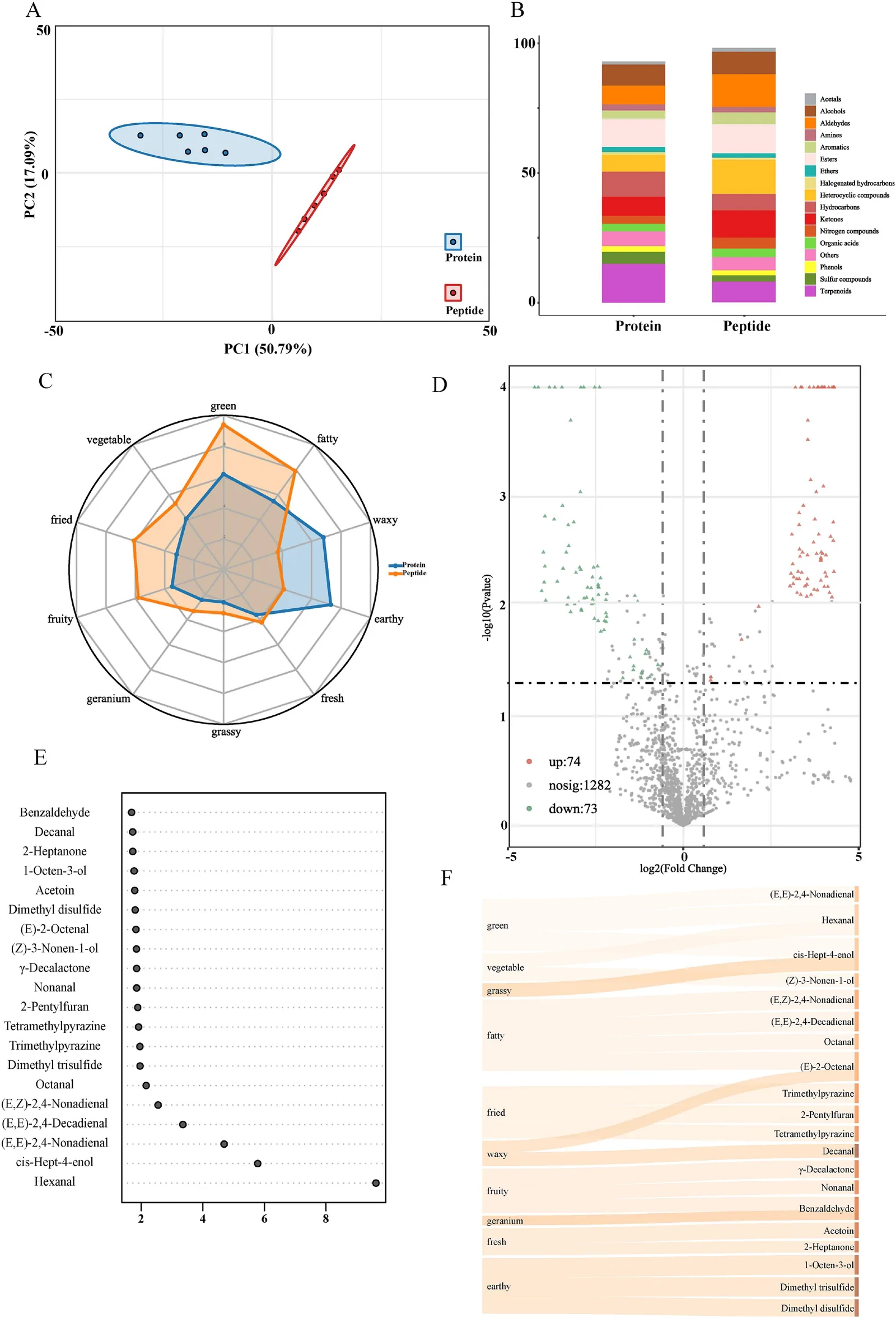
Flavoromics analysis of volatile compounds in sea cucumber protein and hydrolysates. (A) PCA score plot showing discrimination between protein and peptide groups; (B) Compound class distribution of volatiles; (C) Radar plot comparing sensory attribute profiles; (D) Volcano plot displaying differential volatile compounds; (E) Heatmap of selected differential volatiles with clustering analysis; (F) Correlation analysis between key volatile compounds and sensory attributes.

Differential analysis (Fig. 3D-E) identified 147 significantly differential metabolites (VIP > 1, *P* < 0.05), among which 74 were significantly upregulated in the peptide group (up: 74), mainly including hexanal, (E,E)-2,4-nonadienal, (E,E)-2,4-decadienal and other unsaturated fatty acid oxidation products, as well as thermal reaction flavor compounds such as 2-pentylfuran and trimethylpyrazine; 73 compounds were downregulated. Correlation analysis (Fig. 3F) revealed the correspondences between key flavor compounds and sensory attributes: hexanal and (E,E)-2,4-nonadienal were the main drivers of “green” and “fatty” notes, while trimethylpyrazine and 2-pentylfuran were highly correlated with “fried” and “waxy” notes.

Enzymatic hydrolysis is an effective means of regulating sea cucumber flavor and improving its palatability. The loosening of the matrix structure promoted the release of flavor precursors, and new chemical reactions occurred during enzymatic hydrolysis and subsequent drying. Combined with the SEM and spectroscopic results, enzymatic hydrolysis disrupted the dense three-dimensional protein network, making lipids more susceptible to oxygen exposure and enzymatic or auto-oxidation, generating substantial C6-C9 aldehydes (such as hexanal and nonenal). These aldehydes have low olfactory thresholds and serve as the chemical basis for the strong “green” and “fatty” notes of the peptide group (Wang et al., 2019). Furthermore, the enrichment of heterocyclic compounds (such as pyrazines and furans) in the peptide group may be associated with the Maillard reaction (Yu et al., 2026). And the abundant free amino groups and small-molecule peptides generated by enzymatic hydrolysis serve as reactants, reacting with reducing sugars during sample processing (such as heating and drying) to produce volatile compounds with “roasted,” “fried,” and “nutty” aromas, thereby endowing sea cucumber peptides with more complex flavor complexity (Yang et al., 2026).

### 3.9 Identification of sea cucumber peptides

LC-MS/MS analysis revealed the sequence characteristics and physicochemical property distributions of the sea cucumber peptides obtained by enzymatic hydrolysis. As shown in Fig. 4A, the identified peptide lengths were mainly distributed between 5 and 15 amino acids (AA). The peptide-length distribution showed a right-skewed pattern, peaking at 9–10 AA with an average peptide chain length of approximately 9.5 AA, and presenting a long tail extending toward longer peptide sequences. The corresponding molecular weight distribution (Fig. 4C) showed that the majority of peptides were concentrated in the range of 0.5–1.5 kDa, with a peak at approximately 1.0 kDa. In terms of confidence (Fig. 4B), the-10 lgP scores of the peptides were mainly concentrated between 15 and 25, indicating high statistical reliability of the identification results.

**Fig. 4:**
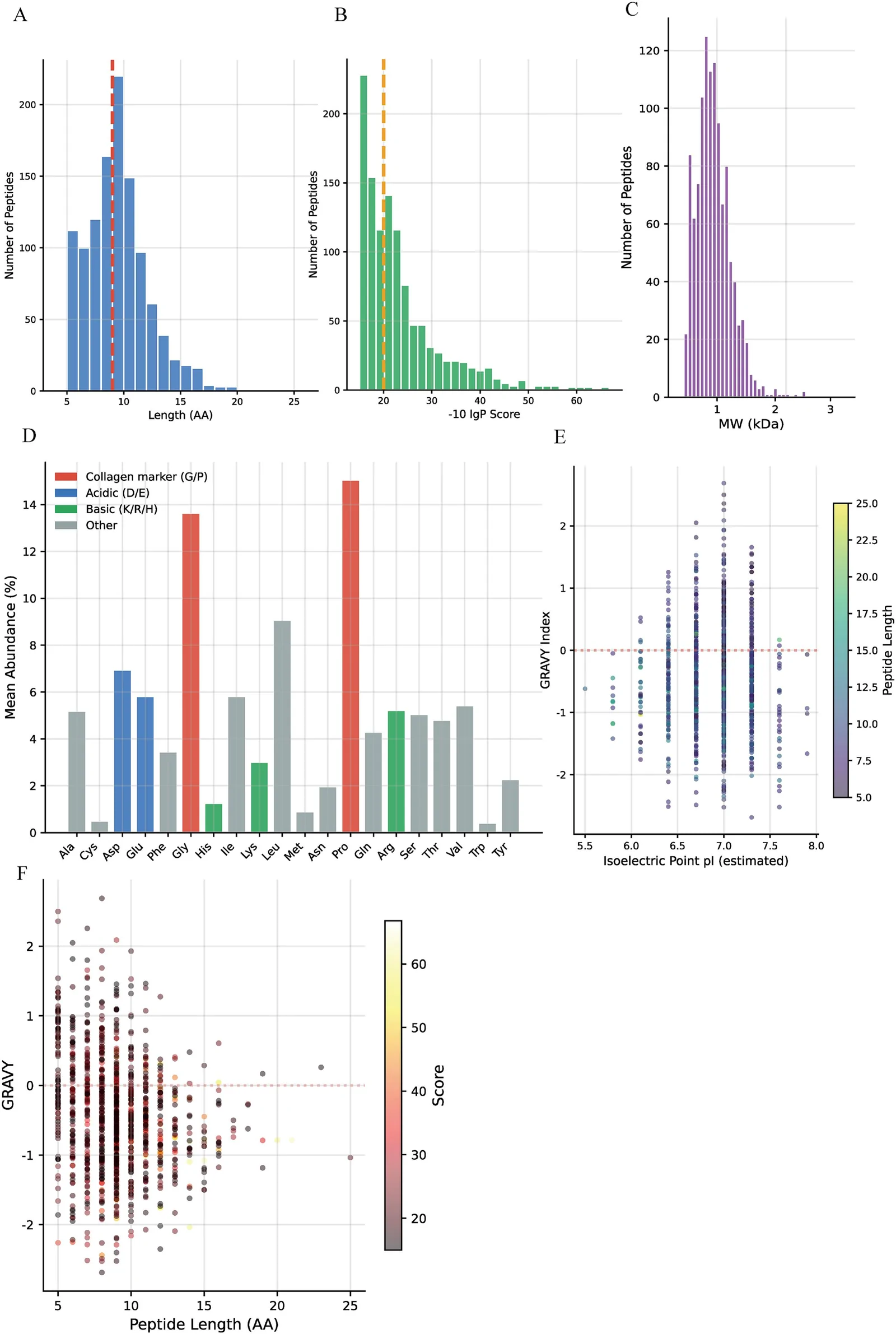
Characterization of peptide sequences identified from sea cucumber hydrolysates by LC-MS/MS. (A) Distribution of peptide lengths; (B) distribution of −10 lgP identification confidence scores; (C) molecular weight distribution; (D) amino acid composition; (E) correlation between isoelectric point (pI) and grand average of hydropathicity (GRAVY); (F) relationship among peptide length, GRAVY value, and identification score.

Amino acid composition analysis (Fig. 4D) revealed that glycine (Gly) and glutamine/glutamic acid (Gln/Glu) were the most abundant residues (both with average abundances exceeding 13%), with glycine as a collagen marker accounting for a very high proportion, confirming that the enzymatic hydrolysis products were predominantly derived from sea cucumber collagen. In addition, acidic amino acids such as aspartic acid/glutamic acid (Asp/Glu) also occupied a considerable proportion. Correlation analysis between isoelectric point (pI) and grand average of hydropathicity (GRAVY) (Fig. 4E) showed that the pI of most peptides was concentrated between 6.0 and 7.5, and the GRAVY values were mostly distributed below 0, suggesting that these peptides were overall weakly acidic to neutral and hydrophilic. Fig. 4F further showed that peptides with high confidence scores (Score > 40) were mainly concentrated in the region of 5–12 AA with negative GRAVY values, indicating that short-chain hydrophilic collagen peptides were the major active forms in the enzymatic hydrolysis products.

The peptide chain length and molecular weight distribution characteristics of the enzymatic hydrolysis products (Fig. 4A, C) directly reflect the cleavage efficiency and substrate accessibility of the specific proteases. The predominance of short peptides around 1 kDa (< 1.5 kDa) facilitates absorption by the human small intestine and provides better solubility and stability, which is of great significance for the development of easily absorbable sea cucumber functional foods. The extremely high proportion of Gly in the amino acid composition (Fig. 4D) is direct evidence of the (Gly-X-Y)n repeating sequence characteristic of marine collagen, which not only validates the purity of the raw material but also suggests that the products may retain collagen-specific bioactivities (such as cytoprotective or moisturizing functions).

From the perspective of physicochemical properties (Fig. 4E-F), enzymatic hydrolysis caused the disintegration of the macromolecular protein structure, exposing a large number of originally buried hydrophilic amino acid residues, resulting in overall hydrophilicity (negative GRAVY values) and weak acidity. This hydrophilic property helps improve the dispersibility of sea cucumber peptides in aqueous systems and reduce precipitation. In addition, the high abundance of free Glu/Asp residues (Fig. 4D) may participate in Maillard reactions during subsequent processing or storage, providing a mechanistic link at the molecular level to the observed increase in roasted aromas (pyrazine compounds) in the flavoromics analysis.

### 3.10 Bioinformatics analysis of sea cucumber peptides

Based on the mass spectrometrically identified sea cucumber peptide sequences, systematic prediction of their potential biological functions was performed through database mapping and bioinformatics analysis.

In Gene Ontology (GO) enrichment analysis (Fig. 5A), the biological process (BP) terms were mainly enriched in “positive regulation of nitric oxide biosynthetic process,” “cellular response to chemical stress,” and “response to organonitrogen compound.” Gene set analysis (Fig. 5B) showed that these enriched pathways mainly involved key functional genes including ERBB2, LYN, CXCR4, ITGB1, and IL1B.

**Fig. 5:**
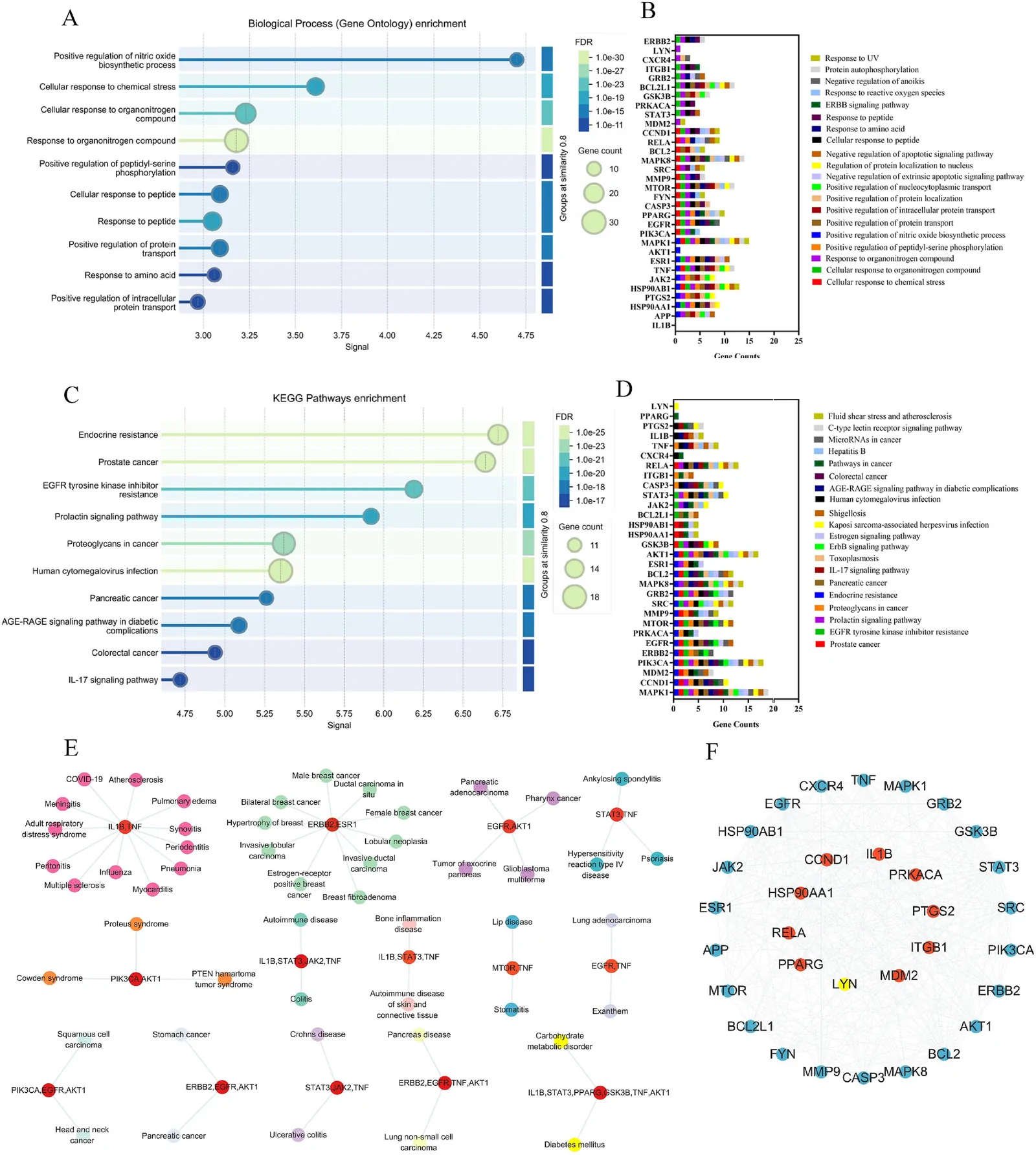
Bioinformatics prediction of the potential functions of sea cucumber peptides. (A) Gene Ontology (GO) enrichment analysis of biological processes; (B) gene set enrichment analysis; (C) KEGG pathway enrichment analysis; (D) pathway-gene network mapping; (E) disease enrichment analysis; (F) protein-protein interaction (PPI) network.

KEGG pathway enrichment analysis (Fig. 5C) showed that the potential targets of sea cucumber peptides were significantly concentrated in “endocrine resistance,” “prostate cancer,” “EGFR tyrosine kinase inhibitor resistance,” and “AGE-RAGE signaling pathway in diabetic complications.” The pathway-gene mapping diagram (Fig. 5D) further revealed that IL1B, TNF, AKT1, STAT3, EGFR, and MAPK1 were core hub genes connecting the above disease pathways.

Disease enrichment analysis (Fig. 5E) showed that sea cucumber peptide targets were closely associated with various inflammatory diseases (such as colitis and periodontitis), metabolic syndromes (atherosclerosis), and malignant tumors (breast cancer and pancreatic cancer). Protein-protein interaction (PPI) network analysis (Fig. 5F) constructed a complex regulatory network with IL1B, TNF, AKT1, STAT3, EGFR, and MAPK1 as core nodes, suggesting that sea cucumber peptides may exert synergistic immunomodulatory or metabolic intervention effects through multi-target and multi-pathway mechanisms.

### 3.11 Screening of sea cucumber peptides

Based on LC-MS/MS identification and bioinformatics prediction, a total of 16 high-confidence sea cucumber-derived peptides were obtained in this study. To screen for candidate peptides with both flavor potential and immunomodulatory activity, the peptides were comprehensively evaluated based on umami probability, bitterness prediction, bioactivity score, anti-inflammatory score, and safety parameters (toxicity, allergenicity, and water solubility). As shown in Supplementary Table 1, FDRGF, CPPGFMG, and FFSLFCLL met the initial screening thresholds of “umami probability ≥ 0.23, bioactivity score ≥ 0.97, non-toxic, and non-bitter” and were selected as core candidates. Among them, FDRGF exhibited the highest umami probability (0.557) and excellent anti-inflammatory activity (0.78), and was non-allergenic. CPPGFMG showed moderate umami probability (0.249) but the highest bioactivity score (0.981). FFSLFCLL possessed both high bioactivity (0.979) and moderate umami potential. The remaining peptides were excluded due to low umami probability (< 0.20), bitterness prediction, poor water solubility, or allergenic risk. Based on these screening results, FDRGF, CPPGFMG, and FFSLFCLL were prioritized as experimental groups for electronic tongue flavor validation to further confirm their taste characteristics.

It should be noted that multiple advanced machine-learning models including TPDM and Umami-YYDS have been proposed for umami peptide prediction in recent studies. Nevertheless, stable public web-servers supporting batch-sequence submission are currently unavailable for these two models. Therefore, the pmlabstack web-server tools were selected for high-throughput preliminary screening of dozens of LC-MS/MS-derived peptide sequences in this work. To mitigate potential prediction bias originating from a single *in silico* tool, electronic tongue assay was further employed for experimental verification of taste characteristics of candidate peptides.

### 3.12 Electronic tongue analysis

Principal component analysis (PCA, Fig. 6A) showed that the three candidate peptides (FDRGF, CPPGFMG, FFSLFCLL) were significantly separated from the blank control (Water) and positive controls (MSG, GSH), with tight clustering of replicate samples within each group, indicating that the electronic tongue could effectively distinguish the flavor profiles of different peptides. Radar plots (Fig. 6B) and bar charts (Fig. 6C) further quantified the specific taste attributes: FDRGF exhibited extremely high umami response (78), second only to MSG (85), with very low bitterness and astringency responses, presenting a clean umami characteristic similar to MSG. CPPGFMG showed moderate umami response (48) but had significant aftertaste and kokumi characteristics, with a flavor profile similar to GSH. In contrast, FFSLFCLL, although possessing some umami, was accompanied by significant bitterness and astringency responses, indicating poor overall taste quality. Heatmap analysis (Fig. 6D) of sensor response differences further corroborated the above flavor distribution characteristics.

**Fig. 6:**
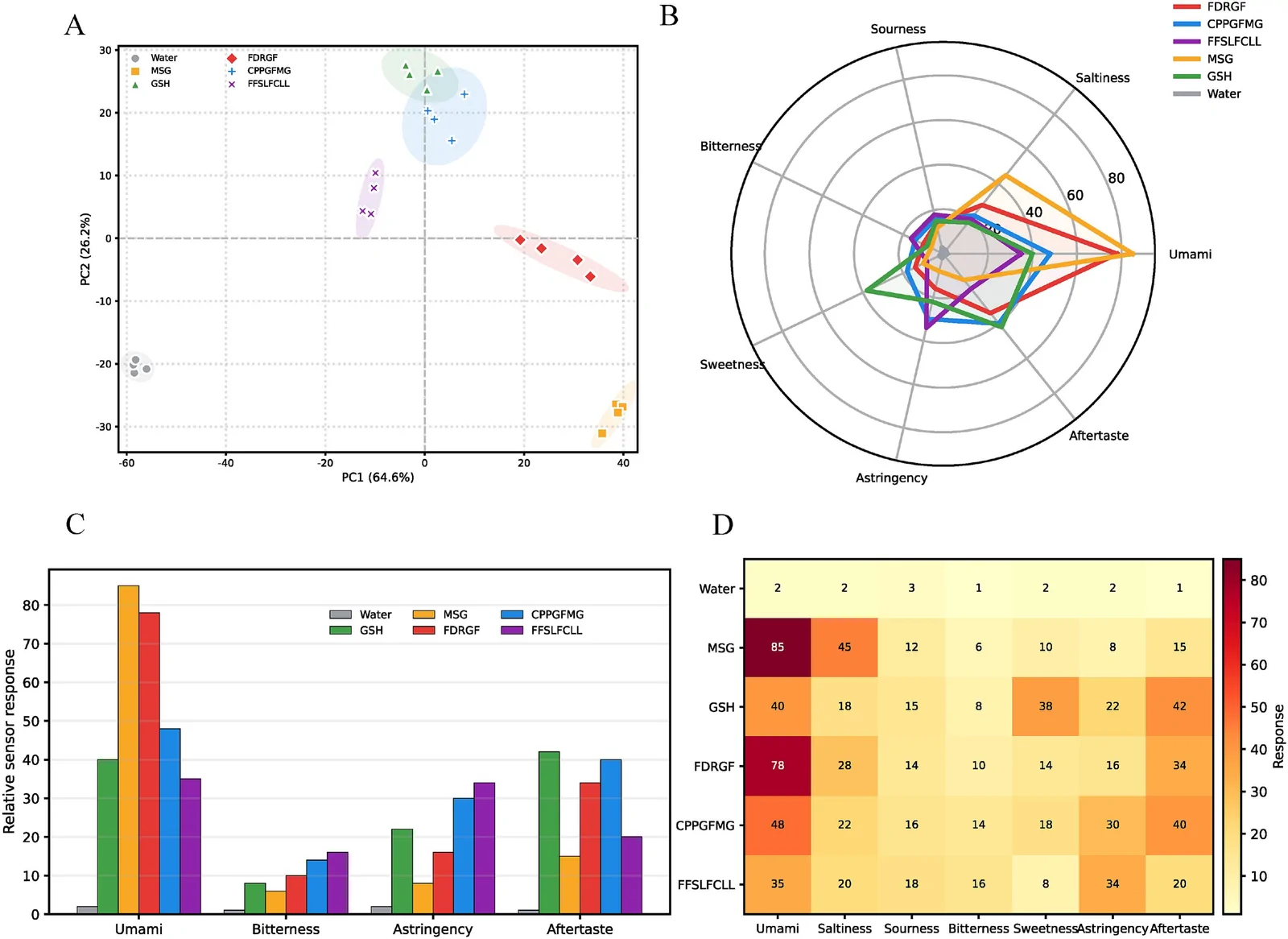
Flavor profile evaluation of candidate peptides by electronic tongue. (A) PCA score plot of three candidate peptides (FDRGF, CPPGFMG, FFSLFCLL) with water (blank), monosodium glutamate (MSG), and glutathione (GSH) as references; (B) radar plot of taste attributes; (C) bar chart of taste response intensities; (D) heatmap of sensor response differences across samples.

The electronic tongue validation results were highly consistent with the previous bioinformatics predictions and clarified the differentiated application potentials of the three peptides. FDRGF, with its high umami probability (0.557) and non-bitter characteristics, exhibited the closest taste performance to commercial monosodium glutamate (MSG) in experiments, and possessed a favorable immunomodulatory/anti-inflammatory score, making it an ideal “umami-immunomodulatory” dual-function core candidate peptide. In summary, the electronic tongue data not only validated the reliability of the bioinformatics screening but also provided a flavor-based rationale for the subsequent preferential synthesis of FDRGF and immunomodulatory activity validation.

### 3.13 Molecular docking and molecular dynamics simulation analysis

In computer-aided bioactive research, molecular docking technology can predict the binding of small molecules to targets, providing critical support for evaluating the functional potential of active substances (Banu et al., 2026). Based on the systematic target screening and analysis results, TNF, AKT1, and IL-1β, which are associated with inflammation, were selected as targets for molecular docking with peptide TH.

Lower negative binding energies indicate stronger binding affinities between active compounds and target proteins. The binding energies of peptide TH with the three targets were −5.3 kcal/mol, −9.9 kcal/mol, and −5.6 kcal/mol, respectively (Fig. 7). The molecular docking results showed that peptide TH exhibited favorable binding affinity to TNF and IL-1β targets, with the strongest binding to AKT1. Given that these three targets are all key nodes in the inflammatory regulatory network, peptide TH most likely exerts its biological effects by binding to these targets, interfering with their biological functions, thereby blocking the initiation and amplification of the inflammatory cascade, ultimately alleviating inflammatory responses and tissue inflammatory damage.

**Fig. 7:**
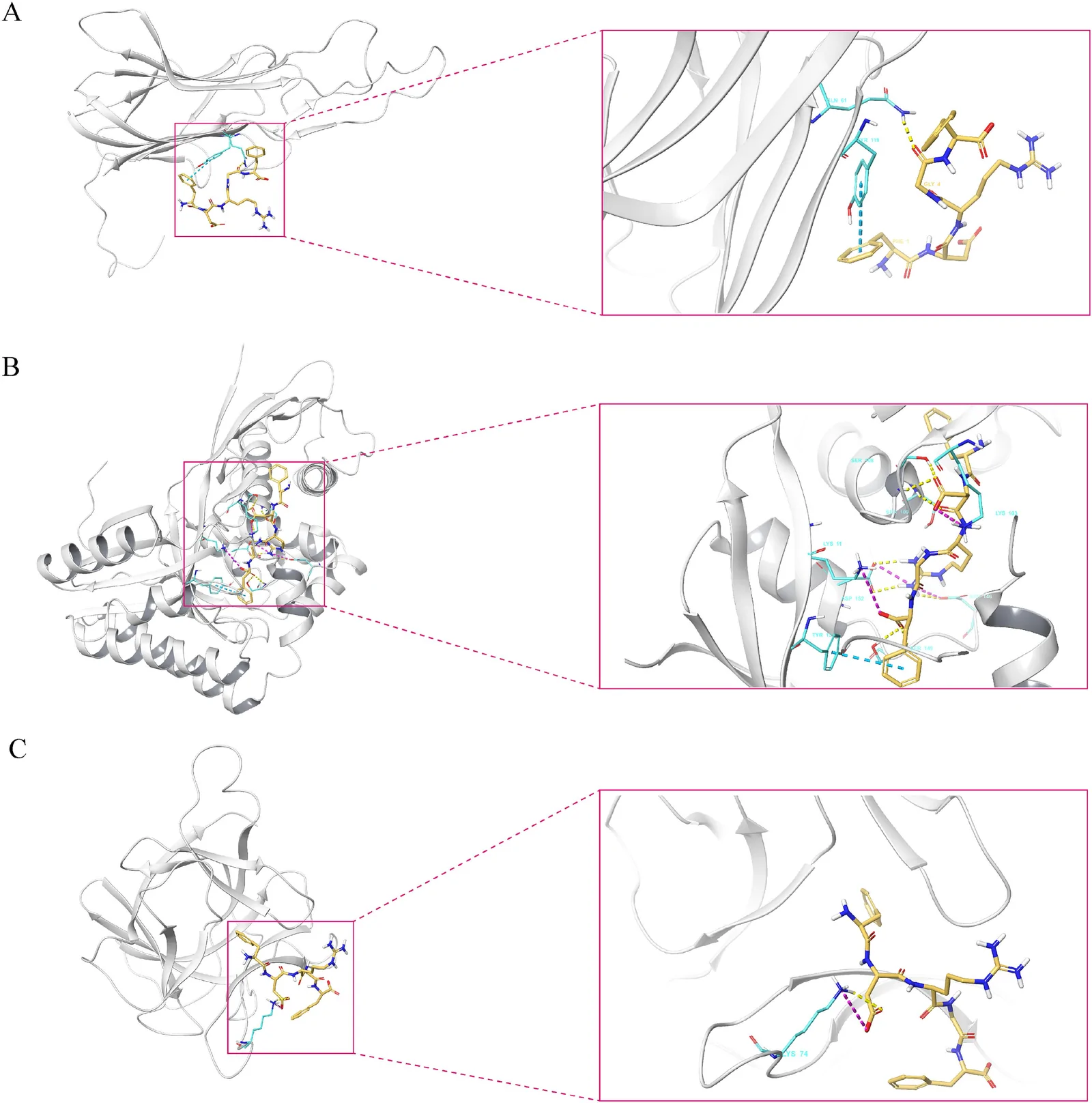
Molecular docking of peptide TH with inflammatory targets. (A) Binding conformation of TH with TNF; (B) binding conformation of TH with AKT1; (C) binding conformation of TH with IL-1β.

In computer-aided drug design, molecular dynamics (MD) simulation is a core technology for analyzing the dynamic behavior of receptor-ligand complexes under physiological conditions. It simulates the atomic motion trajectories of biomolecules and provides quantitative basis for analyzing complex stability and conformational changes, serving as a critical bridge connecting static molecular docking results with *in vivo* dynamic processes (Niazi & Mariam, 2023). In this study, MD simulation was employed, combined with root mean square deviation (RMSD) and root mean square fluctuation (RMSF), to evaluate the overall stability and local residue dynamics of peptide TH complexes with TNF, AKT1, and IL-1β.

RMSD is a key indicator that quantifies the average displacement of specific atoms relative to a reference frame. By monitoring the RMSD values of proteins, conformational change information during simulation can be obtained. As shown in Fig. 8, the RMSD curves of the three protein-ligand complexes (TNF, AKT1, and IL-1β) all converged to stable ranges (1–3 Å), indicating that the systems had reached equilibrium. This result confirmed that peptide TH could maintain stable complex conformations after binding to all three target proteins, which is crucial for regulating the biological functions of the target proteins. It is worth noting that in macromolecular protein simulations, the converged RMSD values should fluctuate steadily within a fixed range; otherwise, it suggests that the system has not reached equilibrium (Kang et al., 2026).

**Fig. 8:**
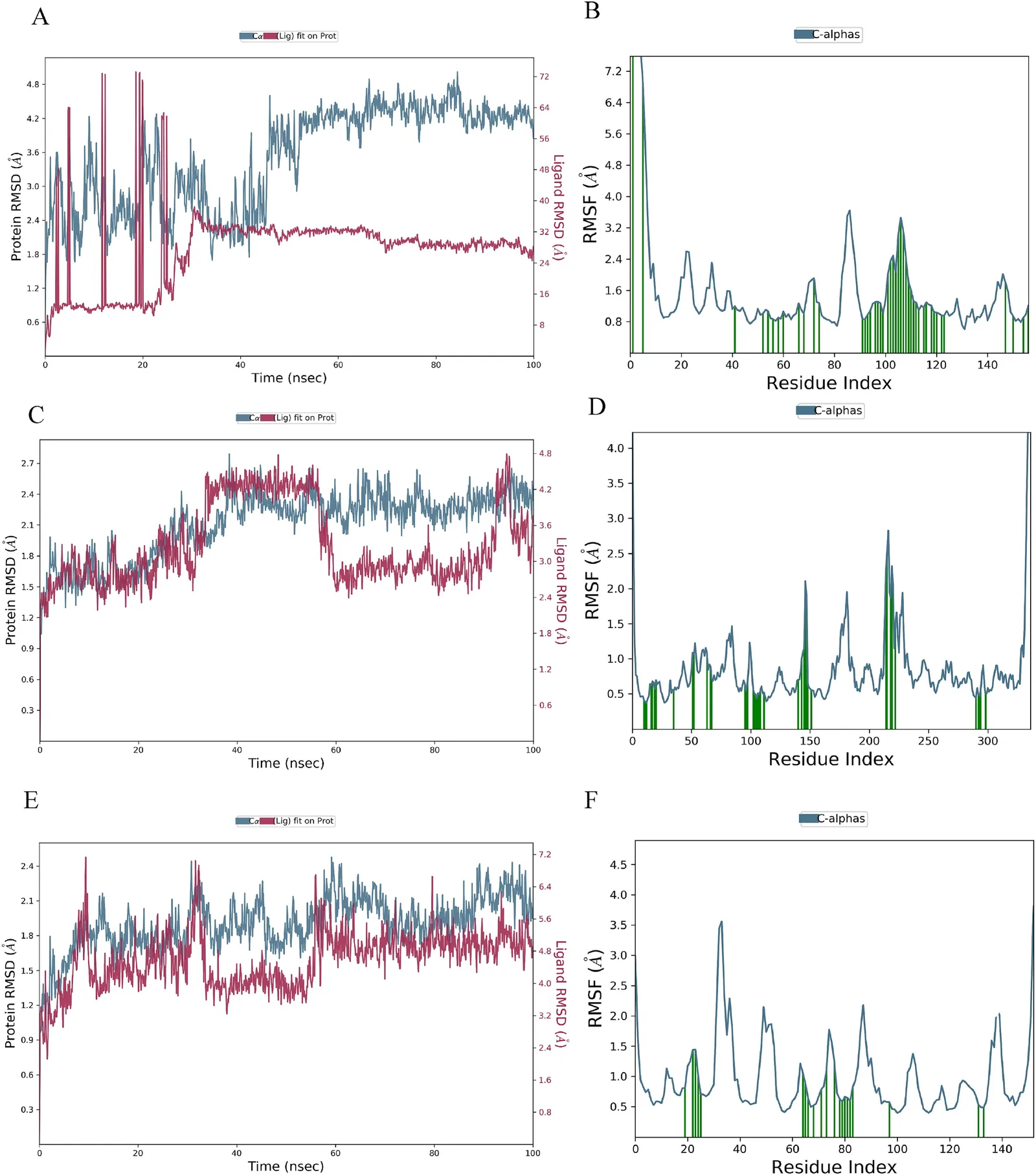
Molecular dynamics simulation of peptide TH bound to inflammatory targets.

RMSF quantitatively evaluates the stability of amino acid residues, reflecting the rigidity characteristics of proteins. The analysis showed that residues in contact with TH exhibited significantly lower RMSF values, indicating that ligand binding restricted the flexibility of these regions, while regions distant from the binding site showed higher fluctuations. This phenomenon was consistently observed across all three target proteins (TNF, AKT1, and IL-1β), suggesting that TH can selectively stabilize its binding sites while maintaining the dynamic properties of other regions of the protein. This binding mode ensures both interaction specificity and the necessary conformational plasticity of the protein, providing a structural basis for developing targeted functional regulation strategies.

In conclusion, the three different target proteins, upon binding with peptide TH, were able to maintain their structural stability in a dynamically changing environment, demonstrating good adaptability and stability.

### 3.14 Immunomodulatory activity validation of TH

The biosafety of TH was evaluated using the MTS assay combined with cell morphology observation. As shown in Fig. 9A, cells treated with different concentrations of TH (6.25–100 μg/mL) maintained good adherence, exhibited healthy morphology, and showed no obvious shrinkage or detachment. Quantitative MTS results (Fig. 9B) showed that cell viability in all treatment groups remained above 95.28%, with no statistically significant differences compared with the blank control group (*P* > 0.05), indicating that TH had no cytotoxicity within the tested concentration range.

**Fig. 9:**
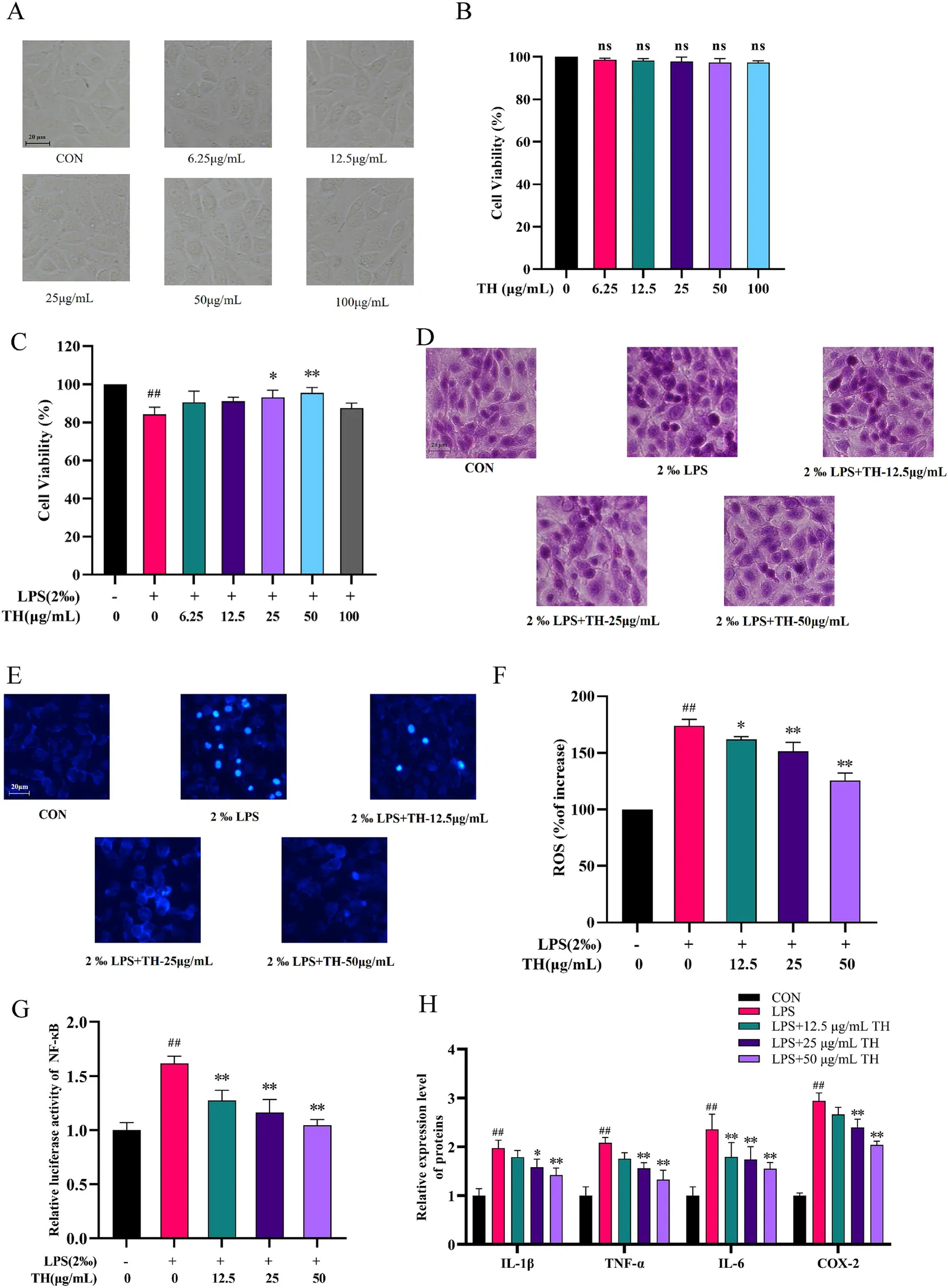
Immunomodulatory activity of peptide TH on LPS-stimulated RAW 264.7 macrophages. (A) Bright-field morphology of cells treated with different concentrations of TH; (B) cell viability detected by MTS assay after treatment with gradient concentrations of TH; (C) cell viability under LPS-induced inflammatory model with TH intervention; (D) H&E staining morphology of cells in each group; (E) Hoechst fluorescence staining of cells in each group; (F) relative level of intracellular ROS in each group; (G) relative transcriptional activity of NF-κB detected by dual-luciferase reporter assay; (H) relative expression levels of inflammation-related proteins IL-1β, TNF-α, IL-6 and COX-2. ##*P* < 0.01 compared with the blank control group; *P* < 0.05, *P* < 0.01 compared with the LPS model group.

In the immunomodulatory activity evaluation (Fig. 9C), LPS stimulation significantly reduced macrophage viability to approximately 84.13% (*P* < 0.01). Pre-treatment with TH (12.5–50 μg/mL) dose-dependently improved cellular metabolic activity detected by MTS assay, reaching a peak at 50 μg/mL (*P* < 0.01). It should be noted that MTS reflects cellular metabolic status rather than complete recovery of immune-cell physiological functions. H&E staining results (Fig. 9D) showed that LPS-treated cells exhibited typical shrinkage, rounding, and detachment. TH treatment groups (especially at 25 and 50 μg/mL) showed significantly improved cell morphology, restoring the typical macrophage-like spreading morphology with observable pseudopodia, suggesting that TH could alleviate LPS-caused morphological damage of macrophages and contribute to the maintenance of cell morphological integrity.

Hoechst fluorescence staining (Fig. 9E) revealed a tendency of mitigation for LPS-triggered nuclear condensation and aberrant nuclear morphology upon TH treatment. Since no quantitative nuclear-morphology scoring was performed, such improvement could only be regarded as qualitative visual observation rather than statistically-valid significant alleviation. Quantitative ROS level detection (Fig. 9F) showed that LPS stimulation significantly increased intracellular ROS levels (*P* < 0.01), while TH treatment dose-dependently reduced ROS levels (*P* < 0.05 or *P* < 0.01), indicating that TH can regulate the oxidative stress levels of immune cells. Dual-luciferase reporter gene assay (Fig. 9G) further confirmed that TH could significantly inhibit LPS-induced NF-κB transcriptional activity in a dose-dependent manner, indicating that TH can regulate the activation status of immune-related signaling pathways. Western blot analysis of immune-related effector molecule expression (Fig. 9H; Fig. S1) showed that LPS stimulation significantly upregulated the protein expression of IL-1β, TNF-α, IL-6, and the inflammatory mediator COX-2. TH pre-treatment dose-dependently downregulated the expression of these immune effector molecules, suggesting that TH can modulate the intensity of macrophage immune responses.

The above results indicate that TH can maintain macrophage homeostasis and modulate immune response intensity through the regulation of ROS and the transcription factor NF-κB. Combined with its previously validated excellent umami taste characteristics, TH exhibited anti-inflammatory potential under LPS-stimulated macrophage inflammatory model, together with favorable umami taste property.

It is worth noting that the immunological-related evaluation in the present study solely relies on the LPS-induced macrophage inflammatory injury *in vitro* model. This model mainly reflects anti-inflammatory performance under inflammatory stress conditions and cannot fully represent comprehensive immunomodulatory effects covering innate-adaptive immune regulation, immune cell differentiation or *in-vivo* immune homeostasis. Besides, the MTS assay only monitors cellular metabolic activity, which cannot be equated to the full recovery of macrophage immune functions. Further investigations including multiple immune cell models and animal *in-vivo* experiments are required to comprehensively evaluate the immunoregulatory potency of peptide TH.

## 4 Conclusion

This study systematically investigated the structural evolution, flavor remodeling, and bioactivity of sea cucumber protein during sequential enzymatic hydrolysis. The results demonstrate that pepsin-trypsin-enterokinase hydrolysis effectively disrupts the compact three-dimensional network of sea cucumber protein, inducing conformational unfolding, increasing the proportion of disordered secondary structures, and exposing aromatic amino acid residues originally buried in the hydrophobic core. The resulting hydrolysates are predominantly composed of short peptides (0.5–1.5 kDa) with enhanced hydrophilicity and improved processing adaptability, as evidenced by the loss of cooperative thermal denaturation behavior. Concurrently, hydrolysis significantly reshapes the volatile flavor profile by promoting lipid oxidation and Maillard reactions, leading to substantial enrichment of aldehydes and heterocyclic compounds that contribute to more complex flavor notes. Through multi-dimensional bioinformatics screening coupled with electronic tongue validation, the TH is identified as a core candidate exhibiting outstanding umami taste, low bitterness, and favorable immunomodulatory potential. Molecular docking and 100 ns molecular dynamics simulations confirm that TH can form stable complexes with key inflammatory targets (TNF, AKT1, and IL-1β), with the strongest affinity observed for AKT1. *In vitro* cellular assays further verify that TH, within the tested concentration range, shows no cytotoxicity and effectively alleviates LPS-induced inflammatory damage by reducing intracellular ROS levels, suppressing NF-κB activation, and downregulating the expression of pro-inflammatory mediators including IL-1β, TNF-α, IL-6, and COX-2. Collectively, these findings elucidate the structure-flavor-activity relationships of sea cucumber protein under multi-enzyme hydrolysis and identify a characteristic umami-tasting peptide possessing *in vitro* anti-inflammatory potential based on LPS-stimulated macrophage model, providing both theoretical insights and experimental evidence for the deep processing of sea cucumber and the development of flavor-functional peptides.

## CRediT authorship contribution statement

**Zhongxing Chu:** Writing – original draft. **Can Long:** Resources. **Kangjie Xu:** Supervision. **Xingjing Zhang:** Methodology. **Dandan Qin:** Investigation. **Zuomin Hu:** Writing – review & editing. **Yaping Zhou:** Project administration. **Feijun Luo:** Writing – review & editing. **Qinlu Lin:** Formal analysis.

## Funding

The authors gratefully acknowledge the funds from the Key Project of State Key R & D Program, China (No. 2022YFF1100200); Young Scientists Fund of the National Natural Science Foundation of China (No. 32603353); the Excellent Youth Project of Hunan Provincial Education Department (No. 25B0319); the Scientific Innovation Fund for Post-graduates of Central South University of Forestry and Technology (No. 2026CX0114).

## Declaration of competing interest

The authors declare that they have no known competing financial interests or personal relationships that could have appeared to influence the work reported in this paper.

## Data Availability

Data will be made available on request.

## References

[bb0005] Ashaolu T.J., Lee C.C., Ashaolu J.O., Tarhan O., Pourjafar H., Jafari S.M. (2024). Pepsin: An excellent proteolytic enzyme for the production of bioactive peptides. Food Reviews International.

[bb0010] Banu N., Alam N., Jhuma S.F., Islam M.N., Alghazali S.A., Awal M.A., Rana M.S. (2026). Chemical profiles, pharmacological insights, and in silico studies of *Antidesma montanum* Blume: An experimental and computer-aided approach. eFood.

[bb0015] Chen X., Qiu Z., Chen J., Ren Y., Liu G. (2026). Recent advances in umami peptides from livestock and poultry by-products: Influencing factors, structural modification, off-flavor control, and bitterness masking. Comprehensive Reviews in Food Science and Food Safety.

[bb0020] Chu Z., Qu G., Yang F., Hu Z., Luo F., Lin Q. (2025). Ultrasound-assisted enzymatic hydrolysis of loach (Misgurnus anguillicaudatus) protein for antioxidant peptides: Process optimization, structural characterization, and Keap1/Nrf2-mediated antioxidant mechanism. Ultrasonics Sonochemistry.

[bb0025] Dou Y., Xia J., Xue M., Liu J., Zhou B., Xu W., Chang Y., Zhan Y. (2026). Dietary mulberry leaf powder supplementation promotes growth of *Apostichopus japonicus* in winter via regulating redox capability, digestion, and intestinal microbiota. Fishes.

[bb0030] Harini R., Natarajan V., Sunil C.K. (2024). Sea cucumber significance: Drying techniques and India’s comprehensive status. Journal of Food Science.

[bb0035] Hidayah N., Manguntungi B., Wahid M., Hansur L., Mustopa A.Z., Irawan H., Meilina L. (2026). Bioconversion of sea cucumber (*Stichopus japonicus*) by *Lactobacillus plantarum*: Enhancement of bioactive peptides, gamma-aminobutyric acid, and corresponding bioactivities. Archives of Microbiology.

[bb0040] Kang C., Chen C.G., Tang C., Arredondo S.C., Zhou M., Fu H., Chipot C. (2026). Convergence is not correctness: Context-dependent performance of enhanced-sampling methods across biological complexity. Nature Communications.

[bb0045] Liang B., Zhang C., Li G., Yang H., Li K., Li R., Liu S., Yu H., Xing R. (2026). Purification and characterization of a newly identified GH18 chitinase BhGH18 from bacillus haynesii and its ultrasound-assisted hydrolysis of chitin. Journal of Agricultural and Food Chemistry.

[bb0050] Liu K., Wang Y., Chang Y., Shi F., Xue C. (2025). A holistic investigation of protein degradation during the autolysis of sea cucumber (Apostichopus japonicus) using a peptidomics strategy. Journal of Agricultural and Food Chemistry.

[bb0055] Lu Z., Sun N., Dong L., Gao Y., Lin S. (2022). Production of bioactive peptides from sea cucumber and its potential health benefits: A comprehensive review. Journal of Agricultural and Food Chemistry.

[bb0060] Maurya D., Mata J.P., Stenzel M.H. (2026). Pathway-dependent formation of compact polypeptide-based single-chain nanoparticles. Macromolecules.

[bb0065] Nain S., Kamboj A., Prabhakar P.K., Chopra R. (2026). Alcalase hydrolysis enhances solubility and foaming properties of perilla protein isolate through structural modification. European Food Research and Technology.

[bb0070] Niazi S.K., Mariam Z. (2023). Computer-aided drug design and drug discovery: A prospective analysis. Pharmaceuticals.

[bb0075] Nikoo M., Guo X., Zhang A., Cui Z., Jiang S., Yang Y., Liu Y., Wang W. (2026). The potential of food-derived bioactive peptides in managing sarcopenia: Identification, structure, mechanism of action, clinical perspective, practical and safety considerations. Trends in Food Science & Technology.

[bb0080] Pang L., Chen C., Liu M., Huang Z., Zhang W., Shi J., Yang X., Jiang Y. (2025). A comprehensive review of effects of ultrasound pretreatment on processing technologies for food allergens: Allergenicity, nutritional value, and technofunctional properties and safety assessment. Comprehensive Reviews in Food Science and Food Safety.

[bb0085] Sospeter E., Ding P. (2026). Untargeted GC–Orbitrap-HRMS fingerprinting reveals tree age-dependent changes in flavor-related metabolites of Musang king durian. Journal of the Science of Food and Agriculture.

[bb0090] Sun M., Pan X., Sun J., Wang W. (2026). Drying method–dependent alterations in protein structure, gastrointestinal digestibility, and peptide profiles in *Penaeus vannamei*. Food and Bioprocess Technology.

[bb0095] Sun X., Udenigwe C.C. (2020). Chemistry and biofunctional significance of bioactive peptide interactions with food and gut components. Journal of Agricultural and Food Chemistry.

[bb0100] Wang D., Su D., Xu H., Chen X., Lv W., Wang Y., Sun X., Guo Y., Ding Y. (2025). New insights into the drying technology of Pleurotus eryngii: Effects on protein structure, properties and product flavor analysis. International Journal of Biological Macromolecules.

[bb0105] Wang Z., Xiao Q., Zhuang J., Feng T., Ho C.-T., Song S. (2019). Characterization of aroma-active compounds in four yeast extracts using instrumental and sensory techniques. Journal of Agricultural and Food Chemistry.

[bb0110] Xiang L., Qiu Z., Zheng Z., Chen H., Qiao Y. (2025). Unlocking the potential of garlic by-product protein: Ultrasound-induced structural modification and fabrication of an ACE inhibitory peptide targeting endothelial dysfunction. Ultrasonics Sonochemistry.

[bb0115] Yang M., Wang J., Shi H., Ma S., Bi Y., Xu X. (2026). From precursors to perception: A unified framework for flavor formation in roasted oilseeds. Journal of the American Oil Chemists' Society.

[bb0120] Yao M., Zhang Y., Yang C., Li T., Liu H., Zhao Y., Memtimin M., Guo S., Duan J.-a., Xiao P. (2026). Novel antioxidant peptides targeting the Keap1-Nrf2 pathway discovered from *Ginkgo biloba* seeds protein hydrolysate: Combining virtual screening, quantum chemical calculation, and zebrafish models in vivo validation. Food Research International.

[bb0125] Yu Y., Nie Q., Chen X., Di Y., Wang A., Yang H., Ren K. (2026). Divergent effects of roasting on the volatile profiles and metabolomes of Pinus pumila and Pinus koraiensis nuts. Food Chemistry: X.

[bb0130] Zhang R., Jia W., Shu J., Zou L., Shi L. (2024). Flavor experiences augmentation strategy for fermented dairy products: Perspective of multimodal perception, starter and enhancer, and processing. Food Reviews International.

[bb0135] Zhang Z., Zhang Y., Zhang M., Yu C., Yang P., Xu M., Ling J., Wu Y., Zhu Z., Chen Y., Shi A., Liu X., Zhang J., Yu P., Zhang D. (2025). Food-derived peptides as novel functional regulation strategies for NLRP3 inflammasome-related diseases: A systematic review. Critical Reviews in Food Science and Nutrition.

