# Supplementary material for: Screening and identification of a novel umami peptide with in vitro anti-inflammatory potential from sea cucumber protein: Integration of flavoromics, bioinformatic prediction, molecular simulation and experimental validation
Source: Food Chem X. 2026 Oct 5;39:104580. doi: 10.1016/j.fochx.2026.104580 (PMC13671053; doi:10.1016/j.fochx.2026.104580)
Supplement: Supplementary file 1 [file mmc1.docx]

| No. | Sequence | Molecular weight | Bioactivity score | Immunity score | water solubility | toxicity | sensitization | Umami Probability | Predicted bitterness | Parent protein source |
| --- | --- | --- | --- | --- | --- | --- | --- | --- | --- | --- |
| 1 | CPPGFMG | 707.88 | 0.98053 | 0.7 | Good | No | No | 0.249 | non-Bitter | Structural/extracellular protein |
| 2 | FFSLFCLL | 988.51 | 0.979033 | 0.56 | Good | No | Yes | 0.23 | non-Bitter | Membrane/defense-related protein |
| 3 | QPPIGCLGMLF | 1174.59 | 0.975371 | 0.93 | Poor | No | No | 0.128 | Bitter | Structural or transmembrane protein |
| 4 | GSPGPPGPPGPAFGRP | 1443.73 | 0.970039 | 0.08 | Good | No | No | 0.042 | Bitter | Type I/body-wall collagen |
| 5 | PQSPFFP | 818.40 | 0.967388 | 0.80 | Poor | No | Yes | 0.062 | Bitter | Collagen-related/extracellular matrix protein |
| 6 | FDRGF | 640.30 | 0.960741 | 0.78 | Good | No | No | 0.557 | non-Bitter | Enzyme/myofibrillar or globin-like protein |
| 7 | QPGPQPLMF | 1013.50 | 0.955188 | 0.86 | Poor | No | Yes | 0.056 | Bitter | Collagen-like/connective tissue protein |
| 8 | GPVGPPGPKGF | 1008.54 | 0.947903 | 0.13 | Poor | No | Yes | 0.042 | Bitter | Sea cucumber collagen |
| 9 | FQPSFIGM | 925.44 | 0.938114 | 0.70 | Poor | No | No | 0.056 | non-Bitter | Enzyme or regulatory protein |
| 10 | GPIGQPGIPGPQGLMG | 1474.76 | 0.935314 | 0.35 | Poor | No | Yes | 0.027 | Bitter | Sea cucumber collagen/gelatin peptide |
| 11 | PDFKF | 652.32 | 0.934389 | 0.84 | Poor | No | No | 0.179 | non-Bitter | Kinase/regulatory protein |
| 12 | FKMPPPP | 812.43 | 0.92473 | 0.70 | Good | No | No | 0.051 | non-Bitter | Cytoskeletal/regulatory protein |
| 13 | GPSFFPGQ | 835.39 | 0.924055 | 0.25 | Poor | No | Yes | 0.045 | non-Bitter | Collagen/connective tissue protein |
| 14 | DAMFFQGL | 927.42 | 0.921004 | 0.66 | Poor | No | Yes | 0.165 | non-Bitter | Lipid-binding/enzyme or myoplasmic protein |
| 15 | GPPGQNGPRGSLGLP | 1402.73 | 0.920206 | 0.04 | Good | No | Yes | 0.022 | Bitter | Sea cucumber collagen |
| 16 | GSPGPPGPPGPA | 986.48 | 0.912456 | 0.13 | Poor | No | No | 0.072 | non-Bitter | Sea cucumber collagen |

Table S1 Physicochemical and flavor properties of candidate peptides from sea cucumber hydrolysates

Figure S1 Effects of sea cucumber peptides on the protein expression of inflammatory factors in LPS-induced cells

Representative protein band images
